# Vasoactive Effects of Chronic Treatment with Fructose and Slow-Releasing H_2_S Donor GYY-4137 in Spontaneously Hypertensive Rats: The Role of Nitroso and Sulfide Signalization

**DOI:** 10.3390/ijms23169215

**Published:** 2022-08-16

**Authors:** Andrea Berenyiova, Martina Cebova, Basak Gunes Aydemir, Samuel Golas, Miroslava Majzunova, Sona Cacanyiova

**Affiliations:** 1 Institute of Normal and Pathological Physiology, Centre of Experimental Medicine, Slovak Academy of Sciences, 841-04 Bratislava, Slovakia; 2 Department of Animal Physiology and Ethology, Faculty of Natural Sciences, Comenius University, 841-04 Bratislava, Slovakia

**Keywords:** spontaneously hypertensive rats, thoracic aorta, mesenteric artery, GYY-4137, fructose

## Abstract

Increased fructose consumption induces metabolic-syndrome-like pathologies and modulates vasoactivity and the participation of nitric oxide (NO) and hydrogen sulfide (H_2_S). We investigated whether a slow-releasing H_2_S donor, GYY-4137, could exert beneficial activity in these conditions. We examined the effect of eight weeks of fructose intake on the blood pressure, biometric parameters, vasoactive responses, and NO and H_2_S pathways in fructose-fed spontaneously hypertensive rats with or without three weeks of GYY-4137 i.p. application. GYY-4137 reduced triacylglycerol levels and blood pressure, but not adiposity, and all were increased by fructose intake. Fructose intake generally enhanced endothelium-dependent vasorelaxation, decreased adrenergic contraction, and increased protein expression of interleukin-6 (IL-6), tumor necrosis factor alpha (TNFα), and concentration of conjugated dienes in the left ventricle (LV). Although GYY-4137 administration did not affect vasorelaxant responses, it restored disturbed contractility, LV oxidative damage and decreased protein expression of TNFα in fructose-fed rats. While the participation of endogenous H_2_S in vasoactive responses was not affected by fructose treatment, the expression of H_2_S-producing enzyme cystathionine β-synthase in the LV was increased, and the stimulation of the NO signaling pathway improved endothelial function in the mesenteric artery. On the other hand, chronic treatment with GYY-4137 increased the expression of H_2_S-producing enzyme cystathionine γ-lyase in the LV and stimulated the beneficial pro-relaxant and anti-contractile activity of endogenous H_2_S in thoracic aorta. Our results suggest that sulfide and nitroso signaling pathways could trigger compensatory vasoactive responses in hypertensive rats with metabolic disorder. A slow H_2_S-releasing donor could partially amend metabolic-related changes and trigger beneficial activity of endogenous H_2_S.

## 1. Introduction

It has been reported that high fructose intake in adult normotensive rats induced insulin resistance, weight gain, and the activation of the renin angiotensin system, resulting in hypertension and metabolic syndrome [1]. Although in our previous study, we did not follow changes characterizing metabolic syndrome after chronic fructose treatment in adult Wistar Kyoto rats, fructose intake impaired the endothelium-dependent vasorelaxation of the thoracic aorta, predicting possible cardiovascular complications. We also demonstrated that chronic fructose intake impaired the synergistic vasomotor manifestation of nitric oxide (NO) and hydrogen sulfide (H_2_S) in these rats [2]. In spontaneously hypertensive rats (SHRs) fed with fructose, an increased level of triacylglycerols in plasma, an additional increase in systolic blood pressure, and kidney and heart hypertrophy were demonstrated; however, significant changes in vasoactive responses of the mesenteric artery were not observed [3].

The position of H_2_S in vasomotor control during arterial hypertension seems to be unique. According to our recent study, H_2_S produced by the vascular wall could represent a form of vasoactive compensatory mechanism to balance impaired vascular tone regulation due to its pro-relaxation effect in SHRs [4]. Moreover, we also demonstrated that SHRs, compared to Wistar rats, revealed an increased vasorelaxant phase of the dual vasoactive responses to exogenously administered H_2_S, which was further potentiated by acute NO deficiency [5,6]. Donors of H_2_S were reported to alleviate pathological changes associated with several types of hypertensions. Sodium hydrosulfide (NaHS) lowered the blood pressure in tail artery of SHRs and was able to delay the switch over from prehypertensive to hypertensive status in this strain [7,8]. Li et al. [9] also demonstrated that chronic i.p. application of NaHS could inhibit oxidative stress and inflammation in hypertension, and then improve endothelial function and ameliorate increased blood pressure. The beneficial effect of NaHS may also be based on its ability to activate Ca^2+^ sparks, spatially and temporally limited Ca^2+^-release events from sarcoplasmic reticulum, in an endothelium-dependent manner to contribute to smooth muscle hyperpolarization and vasodilation [10]. Simple sulfide salts such as NaHS, however, were reported to release H_2_S instantaneously in aqueous solution. Hence, they fail to mimic the biological effect of naturally produced H_2_S, since the relevant tissue comes into contact with high concentrations of H_2_S, the effect of which is time-limited and non-physiological. A slow-releasing H_2_S compound with vasodilator and antihypertensive features, GYY-4137, was suggested as an effective tool with high therapeutic value in cardiovascular disease [11]. Long-term application of this H_2_S donor exhibited a significant decrease in systolic blood pressure and attenuated the myocardial fibrosis in SHRs [12]. Apart from the hypertension-related abnormalities, GYY-4137 could decrease vascular inflammation and oxidative stress, improve endothelial function, and reduce atherosclerotic plaque formation in high fat-fed apolipoprotein E-/- mice [13]. Moreover, it exerted protective effects against high glucose-induced cytotoxicity in the rat cardiac H9c2 cell line [14].

However, data about the effect of increased fructose intake on the vasoactive properties of isolated arteries are limited. In the pathological conditions of essential hypertension, the contractile properties and endothelial function of individual arteries can be modulated differently. Our results on the thoracic aorta of adult SHR showed that adrenergic contractile responses were significantly smaller and endothelial function was only partially impaired, while the NO-dependent component in acetylcholine-induced relaxation as well as NO synthase activity were significantly increased compared to normotensive rats [6]. On the other hand, the mesenteric artery, the second major branch of the abdominal aorta, had a significantly increased contractile response induced by both exogenous norepinephrine and that, released from sympathetic nerve endings. Moreover, endothelial dysfunction was confirmed as well due to a lack of contribution of NO derived from the endothelium, [15,16]. Based on all above-mentioned findings we suggest that the increased fructose consumption could induce metabolic-syndrome-like pathologies in SHRs, and probably differently modulate vasoactive responses of the thoracic aorta and mesenteric artery and the participation of the NO and H_2_S signalizations in these responses. Moreover, a chronic treatment with a slow-releasing H_2_S donor could exert beneficial activity in these conditions. To test these hypotheses, we examined the effect of eight weeks of increased fructose intake on the blood pressure, biometric parameters, vasoactive responses, and NO/NO-synthase (NOS), H_2_S/cystathionine γ-lyase (CSE) signaling pathways, and markers of inflammation and lipid peroxidation in fructose-fed SHRs with or without GYY-4137 chronic application. The present study should answer the question of whether GYY-4137 is a potential tool to diminish the pathological changes in metabolic syndrome.

## 2. Results

### 2.1. General Characteristics of Experimental Animals

A total of 24 rats (8 SHR, 8 SHR+FRU, 8 SHR+FRU+GYY, Table 1) were included in the study and divided into three groups according to long-term fructose intake and treatment with the H_2_S donor, GYY-4137. The chronic fructose intake increased the body weight (BW), heart weight (HW), weight of the retroperitoneal adipose tissue (RTW), length of tibia (TL), and the RTW/TL ratio, which represents body adiposity, but it did not alter the heart trophicity. However, the treatment with GYY-4137 did not affect these parameters. Treatment with both fructose and GYY-4137 had no impact on the plasma level of glucose (GLU), total cholesterol (CHOL), high-density lipoprotein cholesterol (HLD-C), or alanine aminotransferase (ALT). On the other hand, the fructose intake increased the levels of urea and daily fluid intake, but decreased the daily food intake compared to the SHR group. Importantly, chronic fructose intake increased the levels of triacylglycerol (TAG), an effect which was partially attenuated by treatment with GYY-4137. GYY-4137 was able to fully return total protein (TP) and albumin (ALB) levels to those seen in the SHR group, attenuating the significant increase observed upon fructose intake. Interestingly, aspartate aminotransferase (AST) levels were significantly lower in the GYY-4137-treated rats compared to the SHR group, suggesting a possible protective effect against liver pathology. However, no significant change was observed for ALT levels, which would be expected if GYY-4137 had such an effect.

### 2.2. Systolic Blood Pressure in Response to Fructose and GYY-4137 Treatment

**Figure 1:**
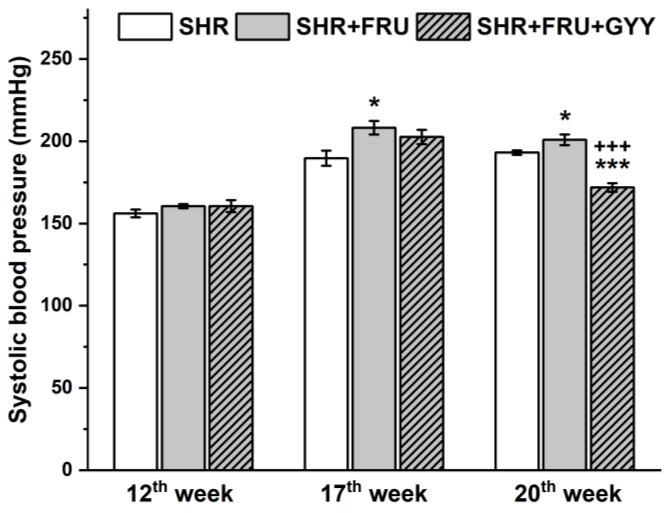
The values of the systolic blood pressure in control SHR (SHR, *n* = 8), SHRs treated with 10% fructose solution (SHR+FRU, *n* = 8), and SHRs treated with 10% fructose solution and 266 µg/kg/day GYY-4137 (SHR+FRU+GYY, *n* = 8). Data are shown as mean SBP ± S.E.M. Statistical analysis was performed by one-way ANOVA with a Bonferroni post-hoc test. * *p* < 0.05 vs. SHR, *** *p* < 0.001 vs. SHR, +++ *p* < 0.001 vs. SHR+FRU.

Systolic blood pressure (SBP) measurements for all treated groups were taken at week 12, week 17 (after 5 weeks of 10% fructose intake), and week 20 (after 8 weeks of fructose intake and 3 weeks of GYY-4137 treatment). Five weeks of fructose intake significantly increased blood pressure in the fructose group compared to control SHR (*p* < 0.05) and tended to increase it in the FRU+GYY group (*p* = 0.058) prior to beginning GYY-4137 treatment. Interestingly, by week 20, SBP was still significantly elevated in the fructose-treated group; however, the blood pressure in the SHRs treated with fructose and GYY-4137 was strongly decreased, even compared to the SHR control group (*p* < 0.001) (Figure 1).

### 2.3. Endothelial Function and Contractile Properties of Isolated Arteries

**Figure 2:**
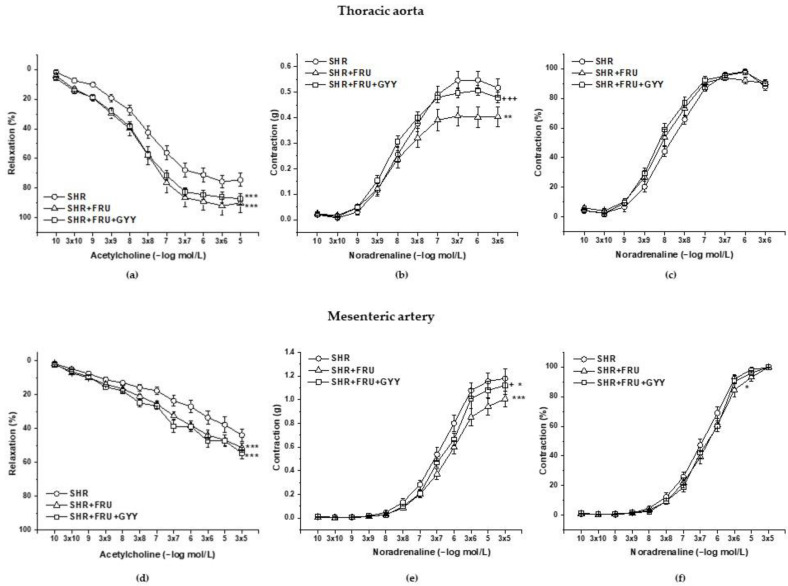
Endothelium-dependent vasorelaxant response of the thoracic aorta (**a**) and mesenteric artery (**d**). Noradrenaline-induced a contractile response of the thoracic aorta (**b**) absolute response, (**c**) percent values of the maximum response), mesenteric artery (**e**) absolute response, (**f**) percent values of the maximum response). Arteries were isolated from spontaneously hypertensive rats (SHRs), SHRs treated with fructose (SHR+FRU), and SHRs treated with fructose and GYY-4137 (SHR+FRU+GYY). The results are presented as the mean ± S.E.M. Statistical analysis was performed by two-way ANOVA with a Bonferroni post-hoc test. * *p* < 0.05, ** *p* < 0.01, and *** *p* < 0.001 vs. SHR, + *p* < 0.05 and +++ *p* < 0.001 vs. SHR+FRU.

Endothelial function was evaluated by the application of cumulative doses of acetylcholine (ACh, 10^−10^ to 10^−5^ moL/L), which induced vasorelaxation mainly mediated by NO. The treatment with both fructose and GYY-4137 had a significant ameliorating effect on the endothelium-derived vasorelaxation of both TA rings (F_(2;585)_ = 42.7; *p* = 5.65 × 10^−18^) (Figure 2a) and MA rings (F_(2;538)_ = 28.9; *p* = 1.32 × 10^−12^) (Figure 2d). Cumulative applications of exogenous noradrenaline (NA, 10^−10^ to 10^−5^ moL/L) generated a vasoconstriction response by activating adrenergic receptors. Chronic fructose intake and GYY-4137 treatment evoked a significant effect in these responses in both TA (F_(2;506)_ = 9.54; *p* = 8.65 × 10^−5^_,_ Figure 2b) and MA (F_(2;538)_ = 13.62; *p* = 1.74 × 10^−6^, Figure 2e). In TA, a decrease in the absolute force of the contractile responses was observed after the fructose intake (*p* < 0.01, Figure 2b); on the other hand, the GYY-437 treatment restored the reduced contractility on the level of the control SHRs (*p* < 0.001, Figure 2b). In MA, the contractile responses were significantly reduced in SHR+FRU group (*p* < 0.001, Figure 2e) and the SHR+FRU+GYY group (*p* < 0.05, Figure 2e) as compared to the control SHR; however, the GYY-4137 treatment was able to partially restore the reduced contractility, which was greater than in the SHR+FRU group (*p* < 0.05, Figure 2e). While in TA, the sensitivity of the adrenergic receptors was not affected by the treatments (Figure 2c), in MA a significant effect was observed (F_(2;538)_ = 3.65; *p* = 0.026, Figure 2f)—the fructose intake shifted the concentration–response curve to the right suggesting the decrease in sensitivity of adrenergic receptors (*p* < 0.05).

### 2.4. Analyses of NO/NOS Pathway

**Figure 3:**
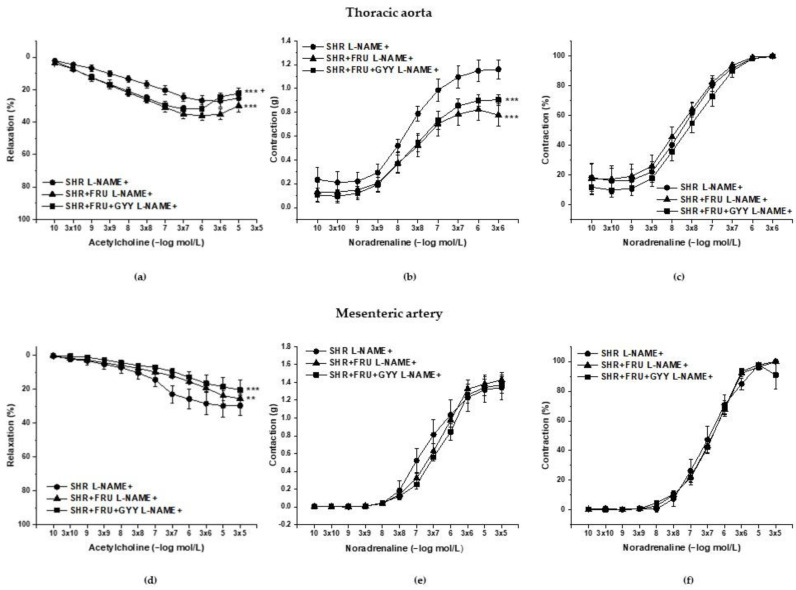
The vasoactive responses of the thoracic aorta (**a**–**c**) and mesenteric artery (**d**–**f**) after incubation with N^G^-nitro-L-arginine methylester (L-NAME). Endothelium-dependent vasorelaxant response of the thoracic aorta (**a**) and mesenteric artery (**d**). Noradrenaline-induced contractile response of the thoracic aorta (**b**)-absolute response, (**c**)-percent values of the maximum response), mesenteric artery (**e**) absolute response, (**f**) percent values of the maximum response). Arteries were isolated from spontaneously hypertensive rats (SHR), SHRs treated with fructose (SHR+FRU) and SHRs treated with fructose and GYY-4137 (SHR+FRU+GYY). The results are presented as the mean ± S.E.M. Statistical analysis was performed by two-way ANOVA with a Bonferroni post-hoc test. ** *p*< 0.01 and *** *p* < 0.001 vs. SHR, + *p* < 0.05 vs. SHR+FRU.

**Figure 4:**
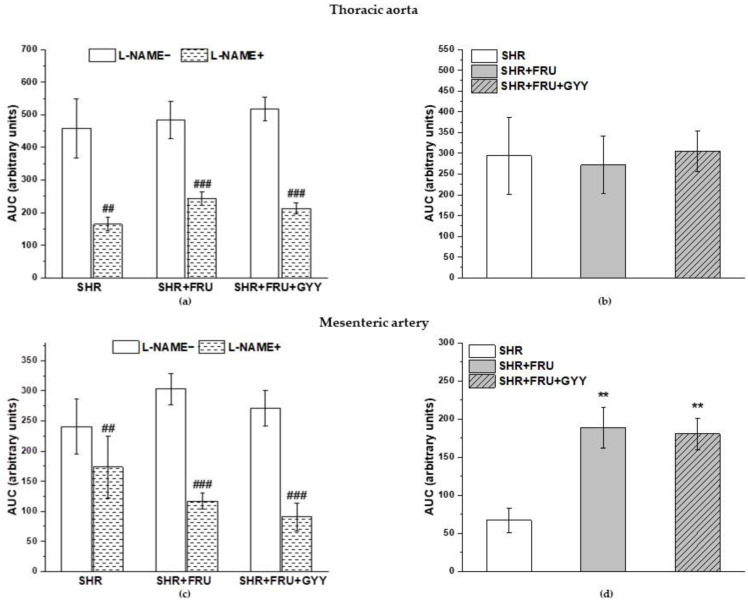
The participation of the NO/NOS system in the relaxant responses of thoracic aorta (**a**,**b**) and mesenteric artery (**c**,**d**). The AUC calculated before and after the pre-treatment with L-NAME in individual groups (**a**,**c**). The difference between the AUC before and after L-NAME—comparison among groups (**b**,**d**). Arteries were isolated from spontaneously hypertensive rats (SHRs), SHRs treated with fructose (SHR+FRU), and SHRs treated with fructose and GYY4137 (SHR+FRU+GYY). AUC—area under the curve, L-NAME—N^G^-nitro-L-arginine methylester. The data are presented as the mean ± S.E.M. Statistical analysis was performed by paired t-test or one-way ANOVA. ^##^ *p* < 0.01, and ^###^ *p* < 0.001 vs. control L-NAME, ** *p* < 0.01 vs. SHR.

The vasoactive manifestation of endogenous NO was investigated via incubation of aortic and mesenteric rings with N^G^-nitro-L-arginine methylester (L-NAME; 10^−4^ moL/L and 10^−5^ moL/L). The concentration-dependent curves after L-NAME incubation are shown in Figure 3. The treatment had a significant effect on the ACh-induced relaxation in TA rings (F_(2;229)_ = 22.9; *p* = 1.18 × 10^−9^); a significant difference was observed not only between the both treated groups (SHR+FRU, SHR+FRU+GYY) (*p* < 0.001) and SHR but between the SHR+FRU and SHR+FRU+GYY groups as well (*p* < 0.05, Figure 3a). Similarly, the used treatments had an effect on the contractile responses of TA (F_(2;202)_ = 19.64; *p* = 2.02 × 10^−8^). The concentration-dependent curves to NA after fructose and GYY-4137 treatments were significantly reduced in comparison with control SHR (*p* < 0.001, Figure 3b). The endothelium-dependent vasorelaxation after L-NAME in MA was affected by the treatments (F_(2;274)_ = 13.6; *p* = 2.54 × 10^−6^), the concentration-dependent curves were the most inhibited in SHR+FRU and SHR+FRU+GYY groups as compared to SHR (Figure 3d). The NA-induced contraction of MA after L-NAME incubation was comparable among the groups (Figure 3e). In both arteries none of the treatments had an impact on the sensitivity of adrenergic receptors to NA (Figure 3c,f). On the other hand, the L-NAME incubation significantly inhibited the endothelium-dependent vasorelaxation in all groups of both TA and MA (Figure 4a,c). While in TA, the participation of the NO component was not affected (Figure 4b), in MA, a significant increase in NO component in the relaxant response was observed in fructose- and GYY-4137-treated groups (*p* < 0.01) (Figure 4d).

**Figure 5:**
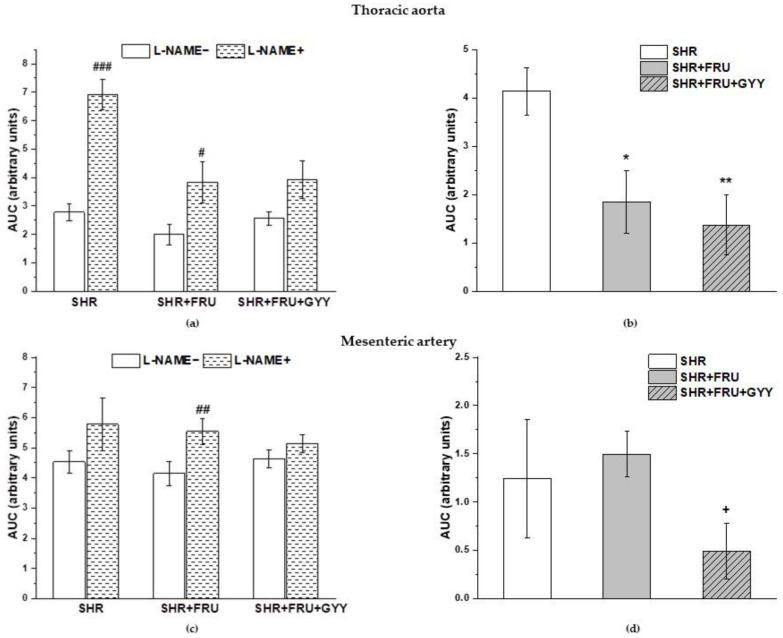
The participation of the NO/NOS system in the contractile responses of thoracic aorta (**a**,**b**) and mesenteric artery (**c**,**d**). The AUC calculated before and after the pre-treatment with L-NAME in individual groups (**a**,**c**). The difference between the AUC before and after L-NAME—comparison among groups (**b**,**d**). Arteries were isolated from spontaneously hypertensive rats (SHRs), SHRs treated with fructose (SHR+FRU), and SHRs treated with fructose and GYY-4137 (SHR+FRU+GYY). AUC—area under the curve; L-NAME—N^G^-nitro-L-arginine methylester. The data are presented as the mean ± S.E.M. Statistical analysis was performed by paired t-test or one-way ANOVA. ^#^ *p* < 0.05, ^##^ *p* < 0.01, and ^###^ *p* < 0.001 vs. control L-NAME-, * *p* < 0.05 and ** *p* < 0.01 vs. SHR, + *p* < 0.05 vs. SHR+FRU.

There was a significant increase after L-NAME incubation in the NA-induced contractile response in the SHR and the SHR+FRU groups, but not in the SHR+FRU+GYY group in the TA (*p* < 0.001, *p* < 0.05, respectively) (Figure 5a). The participation of endo-genous NO in these responses was decreased in the SHR+FRU and SHR+FRU+GYY groups compared to control SHR (Figure 5b). A significant increase in NA-stimulated contractile response after L-NAME incubation was observed in MA only in the fructose-fed group (*p* < 0.01) (Figure 5c), but a significantly decreased participation of NO in contractile response was demonstrated in the group with GYY-4137 treatment as compared to SHR+FRU (Figure 5d).

**Figure 6:**
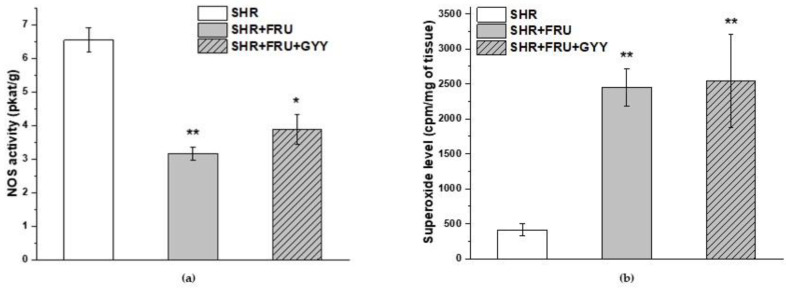
The total NOS activity (**a**) and the superoxide level (**b**) in aortic tissue. Spontaneously hypertensive rats (SHRs), SHRs treated with fructose (SHR+FRU), and SHRs treated with fructose and GYY-4137 (SHR+FRU+GYY). The data are presented as the mean ± S.E.M. Statistical analysis was performed by one-way ANOVA. * *p* < 0.05 and ** *p* < 0.01 vs. SHR.

Total NOS activity in the TA after treatment with both fructose and its combination with GYY-4137 was significantly decreased compared to the SHR group (*p* < 0.01, *p* < 0.05, respectively) (Figure 6a). On the other hand, compared to the SHR group, there was a significant increase in the level of superoxide in both fructose and GYY-4137 groups (*p* < 0.01) (Figure 6b).

**Figure 7:**
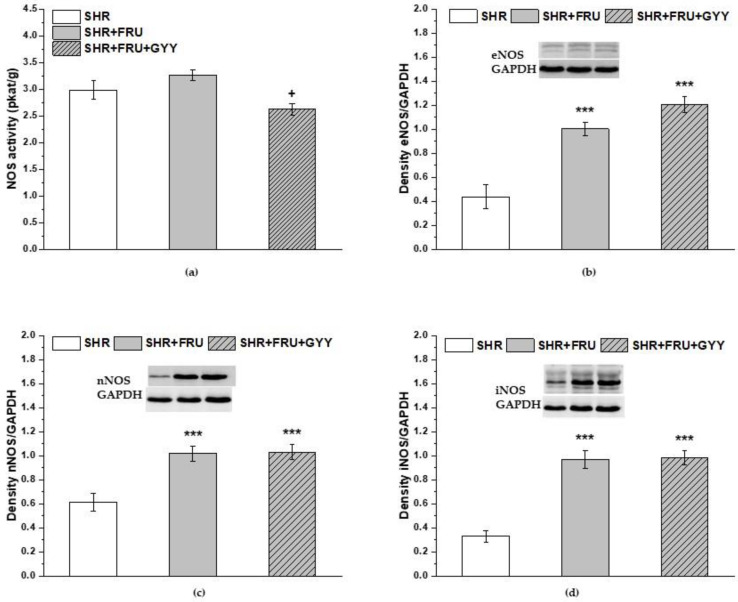
The total NOS activity (**a**), the protein levels of endothelial NOS (**b**), neuronal NOS (**c**), and inducible NOS (**d**) in the left ventricle. Spontaneously hypertensive rats (SHRs), SHRs treated with fructose (SHR+FRU), and SHRs treated with fructose and GYY-4137 (SHR+FRU+GYY). Endothelial NOS—eNOS; neuronal NOS—nNOS; inducible NOS—iNOS. Data are expressed as the mean ± S.E.M. Statistical analysis was performed by one-way ANOVA. *** *p* < 0.001 vs. SHR, + *p* < 0.05 vs. SHR+FRU.

There was a significant decrease in NOS activity in the left ventricle (*p* < 0.05) observed after treatment with GYY-4137 (Figure 7a). The protein levels of endothelial NOS (eNOS), neuronal NOS (nNOS), and inducible NOS (iNOS) in the left ventricle were significantly increased in both the fructose-fed SHRs and GYY-4137-treated groups in comparison to the SHR group (*p* < 0.001) (Figure 7b–d).

### 2.5. Analyses of H_2_S/CSE Pathway

**Figure 8:**
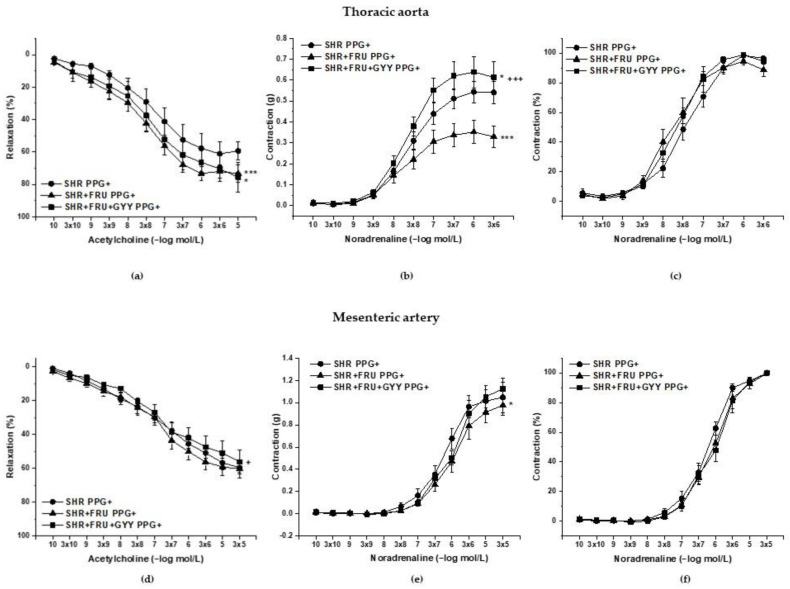
The vasoactive responses of the thoracic aorta (**a**–**c**) and mesenteric artery (**d**–**f**) after incubation with DL-propargylgycine (PPG). Endothelium-dependent vasorelaxant response of the thoracic aorta (**a**) and mesenteric artery (**d**). Noradrenaline-induced contractile response of the thoracic aorta (**b**) absolute response, (**c**) percent values of the maximum response), mesenteric artery (**e**) absolute response, (**f**) percent values of the maximum response). Arteries were isolated from spontaneously hypertensive rats (SHR), SHRs treated with fructose (SHR+FRU) and SHRs treated with fructose and GYY-4137 (SHR+FRU+GYY). The results are presented as the mean ± S.E.M. Statistical analysis was performed by two-way ANOVA with a Bonferroni post-hoc test. * *p* < 0.05 and *** *p* < 0.001 vs. SHR, + *p* <0.05 and +++ *p* < 0.001 vs. SHR+FRU.

**Figure 9:**
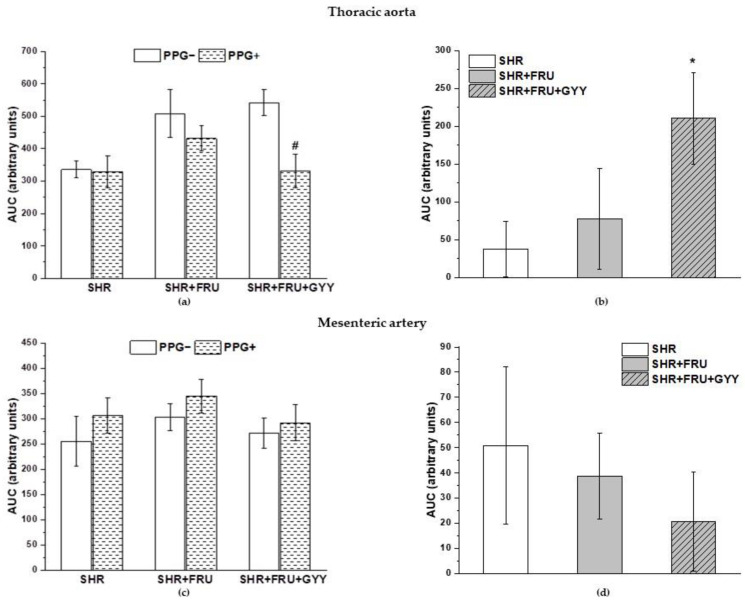
The participation of the H_2_S/CSE system in the relaxant responses of thoracic aorta (**a**,**b**) and mesenteric artery (**c**,**d**). The AUC calculated before and after the pre-treatment with PPG in individual groups (**a**,**c**). The difference between the AUC before and after PPG—comparison among groups (**b**,**d**). Arteries were isolated from spontaneously hypertensive rats (SHRs), SHRs treated with fructose (SHR+FRU), and SHRs treated with fructose and GYY-4137 (SHR+FRU+GYY). AUC—area under the curve; PPG—DL-propargylglycine. The data are presented as the mean ± S.E.M. Statistical analysis was performed by paired *t*-test or one-way ANOVA. * *p* < 0.05 vs. SHR, ^#^ *p* < 0.05 vs. PPG—within the relevant group.

The participation of endogenous H_2_S in relaxant and contractile responses was evaluated by the incubation of both TA and MA rings with the inhibitor of cystathionine-y-lyase (CSE), DL-propargylglycine (PPG, 10 mmoL/L). The concentration-dependent curves after PPG incubation are shown in Figure 8. The treatments had a significant effect on the ACh-induced relaxation after PPG in TA rings (F_(2;185)_ = 9.16; *p* = 1.75 × 10^−4^); a significant difference was observed between the both treated groups (SHR+FRU, SHR+FRU+GYY) (Figure 8a, *p* < 0.001, *p* < 0.05) and SHR. Similarly, the used treatments had an effect on the contractile responses of TA (F_(2;278)_ = 22.74; *p* = 8.39 × 10^−10^). The concentration-dependent response to NA after fructose was significantly reduced in comparison with control SHR (*p* < 0.001), however the GYY-4137 treatment enhanced this response as compared to control (*p* < 0.05) and fructose-fed rats (*p* < 0.001) (Figure 8b). The endothelium-dependent vasorelaxation after PPG incubation was affected by the treatments in MA (F_(2;273)_ = 4.81; *p* = 0.008), and there was a significant difference between the concentration-dependent curves of SHR+FRU and SHR+FRU+GYY groups (*p* < 0.05, Figure 8d). The used treatments had an effect on the NA-induced contraction of MA after PPG incubation (F_(2;274)_ = 3.25; *p* = 0.04), the contractile response in fructose-fed rats was reduced as compared to SHR (Figure 8e). In both arteries, none of the treatments had impact on the sensitivity of adrenergic receptors to NA after PPG (Figure 8c,f). However, PPG incubation significantly inhibited endothelium-dependent vasorelaxation only in groups treated with GYY-4137 in TA (*p* < 0.05) (Figure 9a), and it had no impact on the relaxant responses in MA (Figure 9c). While in TA, the chronic GYY-4137 treatment increased the participation of the endogenous H_2_S component in the relaxation (compared to SHR and SHR+FRU) (Figure 9b), in MA, the participation of H_2_S in vasorelaxant responses was comparable among the groups (Figure 9d).

**Figure 10:**
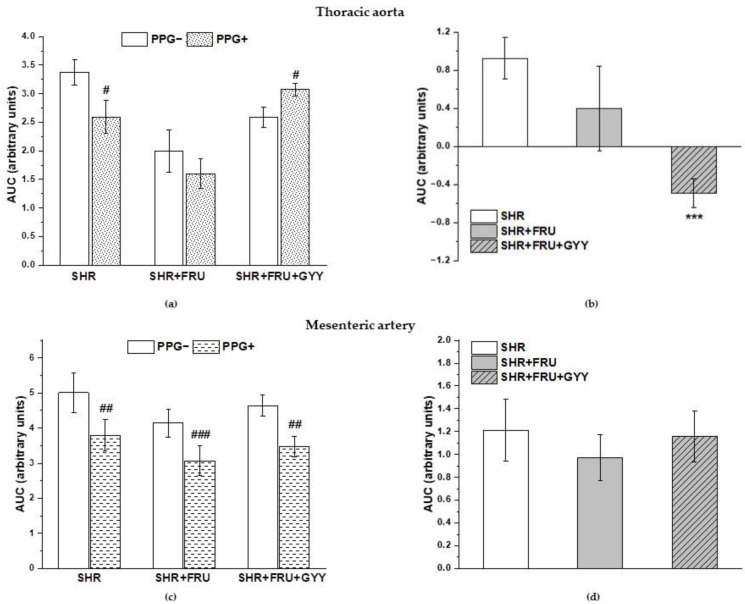
The participation of the H_2_S/CSE system in the contractile responses of thoracic aorta (**a**,**b**) and mesenteric artery (**c**,**d**). The AUC calculated before and after the pre-treatment with PPG in individual groups (**a**,**c**). The difference between the AUC before and after PPG—comparison among groups (**b**,**d**). Arteries were isolated from spontaneously hypertensive rats (SHRs), SHRs treated with fructose (SHR+FRU), and SHRs treated with fructose and GYY-4137 (SHR+FRU+GYY). AUC—area under the curve; PPG—DL-propargylglycine. The data are presented as the mean ± S.E.M. Statistical analysis was performed by paired *t*-test or one-way ANOVA. ^#^ *p* < 0.05, ^##^ *p* < 0.01, and ^###^ *p* < 0.001 vs. control PPG, *** *p* < 0.001 vs. SHR.

PPG incubation significantly decreased the NA-induced contractile response of all groups in MA (*p* < 0.01, *p* < 0.001) (Figure 10c), and the H_2_S component in these responses was unchanged among the groups (Figure 10d). On the other hand, after PPG treatment in the TA, a decrease was observed in the contractile response in the SHR (*p* < 0.05), while there was a significant increase in groups treated with GYY-4137 (*p* < 0.05) (Figure 10a). Importantly, it seems that treatment with GYY-4137 had a significant effect on the vasoactive participation of H_2_S in the contractile responses of the TA compared to the SHR group (*p* < 0.001) (Figure 10b).

**Figure 11:**
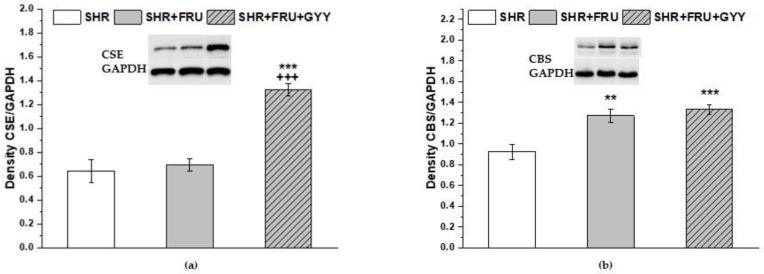
The expression of H_2_S-producing enzymes CSE (**a**) and CBS (**b**) in the left ventricle. Tissue was isolated from spontaneously hypertensive rats (SHRs), SHRs treated with fructose (SHR+FRU), and SHRs treated with fructose and GYY-4137 (SHR+FRU+GYY). CSE—cystathionine γ-lyase; CBS—cystathionine β-synthase. Data are expressed as the mean ± S.E.M. Statistical analysis was performed by one-way ANOVA. ** *p* < 0.01 and *** *p* < 0.001 vs. SHR, +++ *p* < 0.001 vs. SHR+FRU.

The protein expression of H_2_S-producing enzymes CSE and cystathionine β-synthase (CBS) was evaluated in the left ventricle (Figure 11). We observed a significant increase in the CSE expression only in the group treated with GYY-4137 compared to both SHR and fructose intake groups (*p* < 0.001) (Figure 11a). The CBS expression was significantly higher in both groups treated with fructose and GYY-4137 compared to the SHR group (*p* < 0.01, *p* < 0.001, respectively) (Figure 11b).

### 2.6. Markers of Inflammation and Lipid Peroxidation

The protein expression of tumor necrosis factor alpha (TNF-α) and interleukin—6 (IL-6) was evaluated in the left ventricle and aortic tissue (Table 2). In the aortic tissue, there were no differences in protein expression of TNF-α and IL-6 among the experimental groups. We observed a significant increase in the IL-6 (*p* < 0.001) as well as TNFα (*p* < 0.001) in the left ventricle in rats treated with fructose. Whereas the treatment with GYY-4137 reversed the increased expression of TNFα (*p* < 0.01) to the level of the control group, the expression of IL-6 remained increased. Additionally, there were significant increases in conjugated dienes (CD) concentrations in the left ventricle of rats fed with fructose (*p* < 0.01), which were reversed by the treatment with GYY-4137 (*p* < 0.05).

### 2.7. Geometry of the Thoracic Aorta

**Figure 12:**
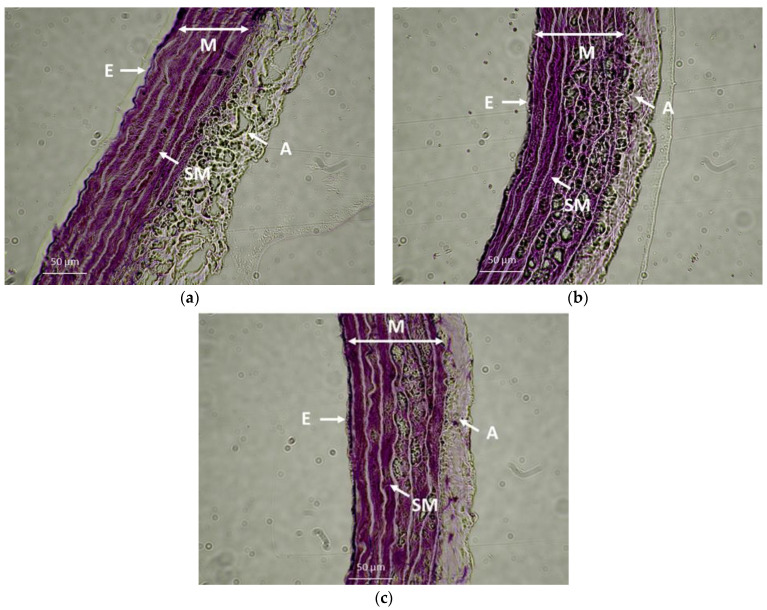
The wall of the thoracic aorta isolated from spontaneously hypertensive rats (SHR) (**a**), SHRs treated with fructose (SHR+FRU) (**b**) and SHRs treated with fructose and GYY-4137 (SHR+FRU+GYY) (**c**). The 5 µm thick cryosections were stained with methylen blue and displayed using optical microscope (40× magnification). E—layer of endothelial cells, SM—layer of smooth muscles, A—adventitia, M—media.

The effect of the increased fructose intake and GYY-4137 treatment on the geometry of the TA is shown in Table 3 and Figure 12. A significant increase in wall thickness was observed in the SHR+FRU and SHR+FRU+GYY groups. The GYY-4137 treatment significantly increased the inner diameter of the TA compared to both SHR and fructose-fed SHR. The cross-sectional area was increased in rats with GYY-4137 treatment as compared to control, but not in rats receiving fructose. The ratio of wall thickness to inner diameter was not changed in any of the experimental groups.

## 3. Discussion

The main findings of the present study are that eight-week fructose treatment triggered metabolic syndrome-related changes in SHRs, and the slow-releasing H_2_S donor, GYY-4137, could partially amend these conditions.

### 3.1. The Effect of Chronic Fructose Intake

Fructose has been considered as a highly lipogenic factor in the diet, and growing evidence suggests its obesogenic role through the formation of substrates for de novo lipogenesis [17]. Hand in hand with this, during fructose consumption, tissues absorb and break down less triacylglycerol-rich lipoproteins, ultimately leading to increased plasma TAG levels [18]. Our results are in line with these findings since the fructose intake evoked an increase in the body adiposity in rats, followed by increased TAG levels in plasma (Table 1). We did not suppose that the increased body weight and lipogenesis might simply be the result of higher calorie consumption. Fructose, which is quickly processed by the liver to glycerol, pyruvate or lactate, and is mostly used for the synthesis of fatty acids, behaves more like a fat than sugar, and even a dose as low as 10% in drinking water can induce signs of metabolic syndrome in animals [19]. Our suggestion is in accordance with the results of Sanguesa et al. [20] who confirmed that although total caloric consumption was higher in glucose-supplemented rats, fructose ingestion had a greater impact in inducing metabolic and aortic dysfunction. Moreover, in fructose fed rats we also confirmed an increase in inflammatory cytokines IL-6 and TNF in cardiac tissue, which was associated with oxidative damage. These findings correspond with suggestions of several authors that fructose induces systemic inflammation and activates inflammatory signalling in local tissues and organs, including the liver, kidneys, gut, and heart [21].

The fructose treatment induced an additional increase in the SBP in fructose-fed rats (Figure 1). Several mechanisms of how fructose consumption can elevate blood pressure have been proposed. Kurbel [22] pointed out that the intake of beverages sweetened with fructose represents a significant osmotic challenge in the body and leads to the retention of large amounts of fluid. Larger trapped fluid volume increases than the renin secretion and enhances sympathetic activity, which in turn stimulates the production of antidiuretic hormone and aldosterone. Subsequently tubules in kidneys may reabsorb more salt and water, and that could lead to increased fluid retention, therefore increasing the volume of body fluids. Moreover, fructose-fed rats in our experiments had a significantly increased fluid intake, proved by the decreased urea level (Table 1), which could strengthen this mechanism. Angiotensin II (Ang II) is also suggested to be associated with fructose-induced hypertension, not only through its vasoconstrictor feature, but it is known to stimulate the generation of reactive oxygen species (ROS) (including superoxide and hydrogen peroxide) [23] and it is able to up-regulate matrix metalloproteinases (MMPs) [24]. MMPs can degrade membrane proteins, which can affect the NO/NOS signalling pathway, since endothelial NOS is a membrane-bound protein. The mentioned mechanisms could explain the decreased total NOS activity and increased superoxide level in TA (Figure 5), demonstrated by our results in SHRs receiving fructose. Furthermore, in pathological conditions, ROS generated by NADPH oxidases can promote eNOS uncoupling resulting in O^2−^ production, rather than NO, and peroxynitrite generation, which leads to the oxidation of tetrabiopterin, limiting the eNOS substrate availability and preventing NO production [25].

Based on the previously mentioned facts, we could expect a decreased NO bioavailability in fructose-fed SHRs, generally associated with impaired relaxant abilities of the vessel wall. However, we observed an enhanced endothelium-dependent relaxation in both TA and MA in these rats. The fructose treatment had a significant effect on the vasorelaxation after L-NAME incubation (Figure 3a) in TA, the augmented response to ACh was NO-independent, since the acute NOS inhibition blocked the vasorelaxation to the same extent in control and fructose-fed rats expressed as the difference of AUC before and after L-NAME incubation. The vasorelaxation in the TA is mainly mediated by NO; however, TA also disposes with other vasorelaxant agents as well, such as prostacyclin and endothelium-derived hyperpolarizing factors (EDHFs), which could serve as reserve relaxant compounds. Several studies reported that in diabetic patients or in obese rats, the NO production is diminished and EDHFs might be able to (at least partially) compensate the decreased NO bioavailability [26,27]. Hydrogen sulfide has been suggested as a possible EDHF in different tissues [28,29]. Our results did not show any changes induced by fructose in the participation of the endogenous H_2_S in the relaxant responses, neither in TA nor in MA, suggesting the manifestation of another signaling compound in the enhanced relaxation of TA. In MA, however, we confirmed that the fructose intake augmented the NO participation in the relaxant responses, which could explain the strengthened vasorelaxation in this artery. According to our results, the chronic fructose consumption did not affect the NO/NOS system in an identical pattern within the cardiovascular system. In contrast to TA, in the left heart ventricle, the total NOS activity was comparable between the control SHRs and those receiving fructose, which was probably caused by an enhanced expression of all NOS isoforms in this tissue (Figure 7). Moreover, Briones et al. [30] demonstrated specifically in the MA tissue of SHR that the expression of the nNOS isoform can be stimulated to counteract endothelial dysfunction and decreased endothelial NO availability, which has been confirmed in MA rings [31]. Based on this, we suggest that the worsening of pathological conditions after fructose administration triggered compensatory stimulation of the NO signaling pathway in certain tissues only (MA and left ventricle but not TA). At the same time, we do not assume the participation of the H_2_S signaling pathway in the modulation of endothelial function induced by fructose intake.

We observed a significantly reduced contractile response to noradrenaline after fructose consumption in both examined arteries. While in TA, the sensitivity of the adrenergic receptors was not affected by the treatment, in MA, we observed a decreased sensitivity in fructose-fed rats, which could be a reason for the reduced contractile response (Figure 2). This response was not associated with stimulated NO/NOS participation, since a decreased and an unchanged NO component was demonstrated in TA and in MA, respectively (Figure 4). Moreover, an unaffected H_2_S participation was observed, as well (Figure 10). We assumed that fructose could reduce the contractile response to noradrenaline by initiating structural remodeling in the vascular wall, since the wall thickness was increased in this group (Table 2). The hypertrophy of the vessel wall does not necessarily mean an increase in the contractile response of the vessel. In our previous study, we demonstrated the hypertrophy of the TA in SHRs; however, the contractile response to noradrenaline was reduced in these rats compared to the normotensive control. We also confirmed that the most increased component participating in hypertrophy of the TA in adult SHRs was the extracellular matrix and not the smooth muscle cells (SMCs) [6]. Other authors also presented that in aortic SMCs, incubated by high fructose, inflammatory mechanisms and remodeling were triggered by activating the nuclear factor kappa B and extracellular signal-regulated kinase 1/2/matrix metalloproteinase-9 pathway in a double-stranded protein kinase (PKR)-dependent manner [32]. Moreover, Zhou et al. [33] found that administration of 10% fructose solution to Sprague Dawley rats caused aortic calcification, increased aortic calcium deposition, and initiated irregular arrangement of elastic fibers in the media of the vascular wall. Taken together, we suppose that the observed compromised contractile function in both vessels is associated with a deteriorating effect of the fructose on the contractile apparatus, rather than representing a beneficial mechanism. Fructose could induce inflammation and structural remodeling (calcification and/or fibrosis) of the arterial wall, which it could lose its elasticity as a result, thereby reducing its contractile capacity.

### 3.2. The Effect of GYY-4137 Treatment

Next, we examined the effect of the slow-releasing H_2_S donor, GYY-4137, on the metabolic and functional changes induced by fructose. While the body adiposity was not changed after the GYY-4137 treatment, GYY-4137 decreased the plasma TAG levels (Table 1). Pan et al. [34] recorded that exposure of 3T3-L1 adipocytes to a high glucose-containing environment increased the TAG content in these adipocytes and a H_2_S donor (NaHS) was able to suppress this process; moreover, it inhibited the aberrant secretion of adipokines in mature adipocytes. The authors suggested that the AMP-activated protein kinase α (AMPKα) may occur downstream of H_2_S signaling, mediating the function of H_2_S in the lipid metabolism of adipocytes.

Three weeks of treatment with a hydrogen sulfide donor was able to reduce the SBP (Figure 1), not only compared to fructose-fed SHRs, but also to control SHRs. The antihypertensive effect of this donor was also observed by Li et al. [10], who reported that two weeks of GYY-4137 administration (133 µmoL/kg i.p.) reversibly decreased the SBP in SHR. This effect occurred within two days and the SBP remained lower for 14 days after finishing the treatment. Similarly, Zhu et al. [35] demonstrated that GYY-4137 was able to lower the SBP in SHR by increasing eNOS expression, reducing oxidative stress, and initiating vascular endothelial cell migration, activated by the vascular endothelial growth factor 2 receptor. In our study, the treatment with GYY-4137 induced an increase in the inner diameter of TA. This effect could contribute to the blood-pressure-lowering effect of GYY-4137, since if lumen of the vessel is increased, the blood brings lower pressure to bear on the vessel wall. Moreover, providing that the increase in SBP after the fructose intake was associated with renin angiotensin system (RAS)-related mechanisms, we could also hypothesize that the GYY-4137-induced SBP decreased by inhibiting the RAS, as we observed in our previous study [36]. We confirmed that a bolus administration of captopril (an angiotensin-converting enzyme inhibitor) in vivo reduced the H_2_S donor-induced decrease in blood pressure. This result suggests that captopril might inhibit the mechanism responsible for the depressor effect of H_2_S. We suggested that captopril disabled the inhibitory effect of H_2_S on RAS, thereby masking the depressor effects of H_2_S [36].

The endothelium-dependent relaxation of both vessels was significantly increased in the group treated with GYY-4137. In this study, we demonstrated that in TA, the GYY-4137 treatment failed to change the participation of endogenous NO in the relaxation, and on the other hand, it enhanced the pro-relaxant effect of endogenous H_2_S (Figure 9). Previously, we confirmed the pro-relaxant effect of H_2_S produced by the vascular wall in SHR, which may represent a compensatory mechanism to offset impaired vascular tone [4]. The exogenous administration of H_2_S could strengthen this effect by stimulation of endogenous H_2_S system. The expression of CSE in the left ventricle was significantly increased, indicating a possibly enhanced H_2_S production. It is well known that H_2_S in a higher concentration acts as a potent vasodilator, predominantly by opening K_ATP_ and K_V_ channels [37]. In addition, as confirmed by our results, GYY administration may specifically affect NOS activity depending on tissue type, as confirmed by its unchanged level in TA and, conversely, its decrease in LV, which, in turn, may modify the interaction between NO and H_2_S in various tissues differently. In MA, the treatment with GYY-4137, similarly to fructose intake, led to the increased participation of NO in the vasorelaxant response. It is possible that although endogenously produced H_2_S did not directly participate in the relaxation of this vessel, its stimulatory effects on the NO pathway may have contributed to the improvement of vasodilator properties in pathological conditions. Our suggestion corresponds with the results of Drucker et al. [38], who showed that GYY-4137 improved mesenteric perfusion and intestinal injury in experimental necrotizing enterocolitis, whereas these benefits were mediated through eNOS-dependent promotion of mesenteric vessels vasodilation.

In both TA and MA, the GYY-4137 ameliorated the contractile responses to NA as compared to fructose-fed rats. In MA, it could be related to the decreased basal NO production, as indicated by the reduced NO participation in the contractile response in this group. This finding seems to contrast with the increased involvement of NO in endothelium-dependent relaxation; however, this discrepancy may again be related to the interaction between NO and H_2_S, which may have a distinct effect in the regulation of different signaling pathways (basal vs. stimulated NO production). As confirmed by the results of our recent study of a non-obese model of metabolic syndrome in hypertriglyceridemic rats, the effect of endogenous H_2_S depended on and was opposite to the type of regulated vasoactive response (contraction vs. relaxation): on one side, H_2_S significantly contributed to the inhibition of vasorelaxation, and on the other side, it showed a significant anticontractile effect [39]. The ability to change the nature of H_2_S action was also confirmed by Emilova et al. [40], who declared the dual vasoactive effect of endogenous H_2_S in rats with streptozotocin-induced diabetes, showing that increasing hyperglycemia was a factor that altered the anti-contractile action of H_2_S to pro-contractile. Our findings from the TA are consistent with these observations, since although GYY-4137 had no effect on NO participation in contractile response compared to fructose-fed rats (Figure 4), it switched the pro-contractile action of endogenous H_2_S to anticontractile. In addition, it has been reported that H_2_S donors ameliorated structural changes in several pathological circumstances. In valvular interstitial cells isolated from samples of human aortic valves, H_2_S donors (including GYY-4137) were able to prevent the calcification of these cells in a concentration-dependent manner [41]. GYY-4137 was also supposed to protect against myocardial fibrosis in SHR [11], which may be related to the inhibition of oxidative stress, the blockage of transforming growth factor β1 (TGF-β1)/Smad2 signaling pathway, and the decrease in the expression of α-smooth muscle actin in cardiac fibroblasts. Moreover, Liu et al. [13] showed that GYY-4137 decreased vascular inflammation and oxidative stress, and reduced atherosclerotic plaque formation in the arterial tissue of pre-clinical model of atherosclerosis, apoE(-/-) mice fed a high-fat diet for four weeks. Although we did not confirm alterations in protein expression of pro-inflammatory cytokines in arterial tissue, we confirmed that the treatment with GYY-4137 reversed fructose-induced oxidative damage and decreased the protein expression of inflammatory marker TNF-α in the left ventricle. This agrees with findings of Li et al. [42] who demonstrated the ability of GYY-4137 to reduce the levels of pro-inflammatory mediators and cytokines such as TNF-α, IL-1β, IL-6 and IL-8 in vivo. Similarly, GYY-4137 has been shown to inhibit lipopolysacharid-evoked release of pro-inflammatory mediators from macrophages or IL-8 secretion and to decrease cell proliferation in airway smooth muscle cells exposed to fetal calf serum growth factor as well as to stimulate the synthesis of the anti-inflammatory chemokine IL-10 in rat plasma during sepsis [43]. Based on these findings, we can assume that the treatment with GYY-4137 could alleviate the fructose-induced structural malformations associated, among others, with the inflammatory process of the tissue and recover the reduced contractile abilities of the vessels.

## 4. Materials and Methods

### 4.1. Guide for the Use and Care of Laboratory Animals

The animals used in the present study were received from the accredited breeding facility of the Center of Experimental Medicine, Slovak Academy of Sciences, Dobra Voda, Slovak Republic. Rats were bred in accordance with the guidelines of the Institute of Normal and Pathological Physiology, Centre of Experimental Medicine Slovak Academy of Sciences (INPP CEM SAS), and were approved by the State Veterinary and Food Administration of the Slovak Republic and by an ethics committee according to the European Convention for the Protection of Vertebrate Animals used for Experimental and Other Scientific Purposes, Directive 2010/63/EU of the European Parliament (permit number: Ro-3888/18-221/3, date 8 January 2019). INPP CEM SAS provided veterinary care. All rats were housed under a 12 h light–12 h dark cycle at constant humidity (45–65%) and temperature (20–22 °C) and had free access to standard laboratory rat chow and drinking water and/or eight weeks of 10% fructose solution.

### 4.2. Experimental Model

Twelve-week-old male SHRs (*n* = 24) were used in this study. The rats were randomly divided into three groups: control spontaneously hypertensive rats (SHR; *n* = 8), fructose-fed SHRs (SHR+FRU; *n* = 8), and fructose-fed SHRs treated with GYY-4137 (SHR+FRU+GYY, *n* = 8). Control SHRs received tap water and fructose-fed groups received 10% fructose solution in drinking water for eight weeks. GYY-4137 (266 µg/kg/day dissolved in 1% dimethyl sulfoxide in physiological solution) was administered intraperitoneally (i.p.) to animals in SHR+FRU+GYY group daily for three weeks long, from 17 to 20 weeks of age. Rats in SHR and SHR+FRU groups received the vehicle i.p. (1% DMSO in physiological solution, volume relevant to actual body weight). All rats were killed at 20 weeks of age.

### 4.3. Blood Pressure, Selected Biometric and Plasma Parameters

Systolic blood pressure (SPB) was measured in pre-warmed rats by non-invasive tail-cuff plethysmography (MRBP, IITC Life Science Inc., Los Angeles, CA, USA) in 12 (beginning of the fructose treatment), 17 (beginning of GYY-4137 administration), and 20 (end of both treatments) weeks of age. All rats were trained for the tail-cuff method of blood pressure determination for two consecutive days before the determination of basal levels of SBP. Five measurements were performed in each rat, and SBP was calculated as the average of the last four measurements.

Rats’ body weight (BW), liquid intake, and food intake were monitored daily during the treatments. The animals were decapitated after brief anesthetization with CO_2_. The final body weight (BW), weight of the heart (HW), weight of the retroperitoneal adipose tissue (RFW), and length of tibia (TL) were measured. The ratios of heart weight to tibia length (HW/TL) and retroperitoneal adipose tissue weight to tibia length (RFW/TL) were calculated to evaluate the degree of cardiac hypertrophy and body adiposity, respectively.

For the determination of the plasma parameters, the trunk blood was collected into pre-prepared heparinized tubes (140 UI/5 mL) and then centrifuged (850× *g*, 10 min, 4 °C, Centrifuge 5430 R, Eppendorf, Hamburg, Germany). The plasma samples were stored at −80 °C until the selected parameters were measured. The levels of glucose (GLU), cholesterol (CHOL), high-density lipoprotein (HLD-C), triacylglycerol (TAG), alanine transaminase (ALT), aspartate transferase (AST), total proteins (TP), albumin (ALB), and urea (UREA) were analyzed by a biochemical analyzer using auxiliary reagent discs (Celercare, MNCHIP Technologies Co., Ltd., Tianjin, China). Next, 100 μL of plasma was pipetted into the sample chamber via the sample port. Then, 430 μL of distilled water was added to the diluent chamber via the diluent port of the test-specific reagent disk.

### 4.4. Vasoactive Responses of Thoracic Aorta and Mesenteric Artery

For the vasoactive examination of the vessels, the descending part of thoracic aorta (TA) and the superior mesenteric artery (MA) were isolated. The vessels were carefully cleaned of connective tissue and cut into rings 3 mm (MA) and 5 mm (TA) in length using a binocular microscope. The rings were vertically fixed between two stainless steel wire triangles and immersed in a 20 mL incubation organ bath with oxygenated (95% O_2_; 5% CO_2_) Krebs solution (118 mmoL/L NaCl; 5 mmoL/L KCl; 25 mmoL/L NaHCO_3_; 1.2 mmoL/L MgSO_4_.7H_2_O; 1.2 mmoL/L KH_2_PO_4_; 2.5 mmoL/L CaCl_2_; 11 mmoL/L glucose; 0.032 mmoL/L CaNa_2_EDTA) kept at 37 °C. The upper triangles were connected to an isometric tension sensor (FSG-01, MDE GmbH., Budapest, Hungary), and the changes in this tension were registered by AD converter SPEL Advanced Kymograph software 4.2.5.0 (MDE GmbH., Budapest, Hungary). A resting tension of 1 g was applied to each ring and maintained throughout a 45–60 min equilibration period.

To test the presence of functional endothelium and the function of vascular smooth muscle cells, a single concentration of acetylcholine (Ach, 10^−5^ moL/L) on noradrenaline (NA, 10^−6^ moL/L, Zentiva, Czech Republic)-precontracted arteries was added. After washing with physiological Krebs solution and an equilibration period, contractile responses to increasing concentrations of exogenous NA (10^−10^–10^−5^ moL/L) were determined in TA and MA. The extent of the contractile responses was expressed as developed changes in isometric tension (g) and as a percentage of the maximum tissue response to the agonist (demonstrating the sensitivity of adrenergic receptors). To examine the endothelium-dependent vasorelaxation, the dose–response curve of Ach (10^−10^–10^−5^ moL/L) was applied on the vascular rings pre-contracted with NA after the achievement of a stabile plateau of the contraction. The rate of relaxation was expressed as a percentage of the NA-induced contraction.

To evaluate the participation of endogenous NO and H_2_S systems in the vasoactive responses of both arteries, the rings were incubated with a non-specific inhibitor of NOS, i.e., N^G^-nitro-L-arginine methyl ester (L-NAME, 10^−4^ moL/L in TA and 10^−5^ moL/L in MA), or an inhibitor of cystathionine-y-lyase (CSE), i.e., DL-propargylglycine (PPG, 10 mmoL/L). All drugs were acutely incubated for 20 min in the organ bath, and the concertation–response curves to NA and Ach were repeated. The concentration-dependent curves (to ACh and NA) after the incubation with the inhibitors were expressed to compare the extension of the NO and H_2_S inhibition among the experimental groups. To express the NO and H_2_S participation in the vasoactive responses in each group, areas under curves (AUC, in arbitrary units) were calculated and compared before and after incubation with the inhibitors.

### 4.5. Measurement of Superoxide Production in Selected Tissues

The level of superoxide anions was measured by a chemiluminescent method using lucigenin based on the intensity of the emitted photons. The TA samples were placed in Krebs solution immediately after removal. Oxygenated (mixed 95% O_2_, 5% CO_2_) 50 μmoL/L lucigenin and samples in oxygenated Krebs solution were incubated in the dark for 20 min at 37 °C. After incubation, either background chemiluminescence or chemiluminescence produced by the used tissues was measured using a TriCarb 2910TR liquid scintillation analyzer (PerkinElmer, Waltham, MA, USA). The resulting mean values were expressed as cpm/mg (count per minute/milligram) of tissue.

### 4.6. Total NO Synthase Activity

Total NOS activity was determined in crude homogenates of the left ventricle aorta by measuring [3^H^]-L-citrulline formation from [3^H^]-L-arginine (MP Biochemicals, Santa Ana, CA, USA), as described elsewhere [44]. [3^H^]-L-citrulline was measured with the Quanta Smart TriCarb Liquid Scintillation Analyzer (TriCarb, Packard, UK). NOS activity is expressed as picokatal per gram of protein (pkat/g protein).

### 4.7. Western Blotting

Protein expressions of endothelial, neuronal, and inducible NOS isoforms, CSE, CBS and inflammatory markers tumor necrosis factor alpha (TNF-α) and interleukin-6 (IL-6) were determined in the left ventricle by Western blot analysis. Briefly, tissue samples of the left ventricle were homogenized in lysis buffer (0.05 mM Tris containing protease inhibitor cocktail, Sigma-Aldrich, Germany). After centrifugation (15,000 rpm at 4 °C for 20 min), protein concentrations were determined by Lowry assay. Supernatants were subjected to SDS-PAGE using 12% or 15% gels to examine protein and transferred to nitrocellulose membranes. Membranes were blocked with 5% non-fat milk in Tris buffer solution (TBS; pH 7.6) containing 0.1% Tween-20 (TBS-T) for 1 h at room temperature and probed with a primary polyclonal rabbit anti-endothelial NOS, anti-neuronal NOS and GAPDH (Abcam, Cambridge, UK), rabbit polyclonal anti-inducible NOS (BioRad, Inc., Hercules, CA, USA), and rabbit polyclonal anti-CBS, mouse monoclonal anti-CSE antibodies mouse monoclonal anti-TNF-α antibodies (Proteintech, Manchester, UK) and rabbit polyclonal anti-IL-6 antibodies (Bioss Antibodies, Woburn, MA, USA) (overnight at 4 °C. Antibodies were detected using a secondary peroxidase-conjugated anti-rabbit antibody (Abcam, Cambridge, UK) or anti-mouse antibody (Cell Signalling, Danvers, MA, USA) at room temperature for 2 h. The intensity of bands was visualized using the enhanced chemiluminescence system (ECL, Amersham, UK), quantified by using the Chemi-DocTM Touch Imagine System (Image LabTM Touch software 2.4 BioRad, Inc., Hercules, CA, USA) and normalized to GAPDH bands.

### 4.8. Morphological Study

TAs were harvested, cleaned of connective tissue in Krebs solution, and immediately embedded in OCT compound (Tissue-Tek, Sakura Finetek, Torrance, CA, USA) and stored at −80 °C. Frozen sections were cut with a cryostat (Leica CM 1860 UV, Leica Biosystem, Deer Park, IL, USA) and 5 μm thick slices were cut perpendicularly to the long axis, collected on Superfrost Plus slides, and methylene blue staining was performed. Both the wall thickness (tunica media+intima) and inner circumference were measured by light microscopy (Primostar 3, Carl Zeiss Microscopy GmbH., Jena, Germany). The wall thickness (WT) was measured at 45° intervals around the circumference of the artery. For the morphometric determination of TA, the inner diameter (ID), cross-sectional area (CSA) of tunica intima and tunica media, and wall thickness/inner diameter ratio (WD) were evaluated from the measured data, and mean values were calculated.

### 4.9. Determination of Conjugated Dienes Concentration (CD)

The concentration of conjugated dienes as a marker of lipid peroxidation was measured in order to determine levels of oxidative stress occurring in organs of treated animals as described elsewhere [45]. Briefly, samples from the left ventricle and the liver were homogenized in 15 mmoL/L EDTA containing 4% NaCl. Lipids were extracted using mixture of chloroform and methanol in 1:1 ratio. Chloroform was evaporated in N2 atmosphere. After the addition of cyclohexane, the absorbance was determined (NanoDrop OneC, UV-Vis spectrophotometer; Thermo Fisher Scientific, Waltham, MA, USA). The concentration of CD was calculated using the extinction coefficient ε = 29,000 L/moL/cm and expressed as μmoL per g tissue.

c = (χ × 1000)/m [mmoL/g tissue] χ = 1000 × (A/5.8)

### 4.10. Statistical Analysis

The data were expressed as the mean ± S.E.M. For the statistical evaluation of vasoactive responses between groups, two-way analysis of variance (ANOVA) and the Bonferroni post-hoc test were used. To evaluate general cardiovascular parameters, plasmatic parameters, AUC before and after inhibitors, NO synthase activity, and superoxide levels, Student’s *t*-test and one-way ANOVA were used. Differences between means were considered significant at *p* < 0.05. Data were analyzed with OriginPro 2019b (OriginLab Corporation, Northampton, MA, USA).

### 4.11. Drugs

All the chemicals used in this study were purchased from Sigma-Aldrich unless otherwise stated.

## 5. Conclusions

Our results suggest that sulfide and nitroso signaling pathways could trigger compensatory vasoactive responses in hypertensive rats with metabolic disorder. Unlike in thoracic aorta, where another factor acted, in the mesenteric artery, fructose intake triggered compensatory action of the NO signal pathway. On the other hand, although the slow H_2_S-releasing donor generally recovered contractility altered by fructose intake, it stimulated beneficial pro-relaxant and anti-contractile action of the endogenous sulfide pathway in the thoracic aorta.

## Figures and Tables

**Table 1:** General characteristics of experimental animals.

Parameter	SHR	SHR+FRU	SHR+FRU+GYY
*n*	8	8	8
BW (g)	313 ± 3.5	333.6 ± 5.6 **	331.9 ± 6 *
HW (g)	1.095 ± 0.05	1.254 ± 0.16 *	1.232 ± 0.15 *
RTW (g)	2.03 ± 0.17	2.96 ± 0.16 **	2.98 ± 0.14 ***
TL (mm)	34.79 ± 0.24	36.93 ± 0.34 **	36.98 ± 0.42 ***
HW/TL (mg/mm)	28.71 ± 3.18	33.99 ± 0.48	33.34 ± 0.33
RTW/TL (mg/mm)	58.33 ± 4.98	79.91 ± 3.93 **	80.74 ± 3.93 **
GLU (mmoL/L)	8.26 ± 0.35	9.04 ± 0.77	8.14 ± 0.43
CHOL (mmoL/L)	2.67 ± 0.19	2.71 ± 0.22	2.41 ± 0.13
HLD-C (mmoL/L)	1.65 ± 0.09	1.9 ± 0.17	1.6 ± 0.09
TAG (mmoL/L)	1.2 ± 0.07	2.2 ± 0.24 **	1.67 ± 0.08 **^+^
ALT (U/L)	80.86 ± 4.34	86.14 ±7.48	82.75 ±9.43
AST (U/L)	221.14 ± 14.92	198.14 ± 18.21	177.63 ± 8.57 *
TP (g/L)	74.43 ± 2.35	84.37 ± 3.86 *	75.1 ± 2.2 ^+^
ALB (g/L)	45.56 ± 1.11	50.17 ±1.79 *	45.7 ± 1.09 ^+^
UREA (mmoL/L)	7.86 ± 0.11	5.18 ± 0.47 ***	4.95 ± 0.51 ***
Fluid intake (mL/day)	33.45 ± 2.72	79.41 ± 2.45 ***	73.8 ± 1.19 ***
Food intake (g/day)	21.1 ± 0.86	16 ± 1.19 *	16.49 ± 1.77 *

Abbreviations: *n*—number of rats; SHR—spontaneously hypertensive rats; SHR+FRU—spontaneously hypertensive rats treated with 10% fructose solution; SHR+FRU+GYY—spontaneously hypertensive rats treated with 10% fructose solution and H_2_S donor GYY-4137; BW—body weight; HW—heart weight; RTW—retroperitoneal adipose tissue weight; TL—tibia length; HW/TL—ratio of heart weight to tibia length; RTW/TL—ratio of retroperitoneal adipose tissue weight to tibia length; GLU—glucose; CHOL—total cholesterol; HDL-C—high-density lipoprotein cholesterol; TAG—triacylglycerol; ALT—alanine aminotransferase; AST—aspartate aminotransferase; TP—total protein; ALB—albumin. Values are the mean ± S.E.M. Statistical analysis was performed by one-way ANOVA with a Bonferroni post-hoc test; * *p* < 0.05, ** *p* < 0.01, and *** *p* < 0.001 vs. SHR, ^+^ *p* < 0.05 vs. SHR+FRU.

**Table 2:** Protein expression of IL-6 and TNFα and Conjugated Dienes Concentration.

Parameter	SHR	SHR+FRU	SHR+FRU+GYY
IL-6 (aorta) (Density IL-6/β-actin)	0.078 ± 0.02	0.085 ± 0.03	0.102 ± 0.02
IL-6 (left ventricle) (Density IL-6/GAPDH)	0.305 ± 0.02	0.570 ± 0.04 ***	0.616 ± 0.04 ***
TNFα (aorta) (Density TNFα/β-actin)	4.893 ± 0.35	5.221 ± 0.25	4.129 ± 0.39
TNFα (left ventricle) (Density TNFα/GAPDH)	0.108 ± 0.02	0.316 ± 0.05 ***	0.160 ± 0.03 ^++^
CD left ventricle (nmol/g)	1493.79 ± 125.44	2815.33 ± 211.93 **	1808.37 ± 140.49 ^+^

Abbreviations: IL-6—interleukin -6; TNFα—tumor necrosis factor alpha; CD—conjugated dienes; Statistical analysis was performed by one-way ANOVA with a Bonferroni post-hoc test; ** *p* < 0.01 and *** *p* < 0.001 vs. SHR, ^+^ *p* < 0.05 and ^++^ *p* < 0.01 vs. SHR+FRU.

**Table 3:** Geometry of the thoracic aorta.

Parameter	SHR	SHR+FRU	SHR+FRU+GYY
WT (µm)	87.88 ± 2.06	101.66 ± 3.87 **	102.41 ± 2.16 **
ID (µm)	1655.51 ± 51.74	1682.49 ± 65.38	2064.83 ± 47.31 *** ^++^
CSA (µm^2^) × 10^3^	404.93 ± 21.2	568.57 ± 23.36	639.75 ± 3.71 **
WD (WT/ID) × 100	5.34 ± 0.56	6.09 ± 0.37	5.33 ± 0.31

Abbreviations: SHR—spontaneously hypertensive rats; SHR+FRU—spontaneously hypertensive rats treated with 10% fructose solution; SHR+FRU+GYY—spontaneously hypertensive rats treated with 10% fructose solution and H_2_S donor GYY-4137; WT—wall thickness; ID—inner diameter; CSA—cross-sectional area; WD—wall thickness/inner diameter ratio. Values are the mean ± S.E.M. Statistical analysis was performed by one-way ANOVA with a Bonferroni post-hoc test; ** *p* < 0.01 and *** *p* < 0.001 vs. SHR, ^++^ *p* < 0.01 vs. SHR+FRU.

## Data Availability

The data presented in this study are available on request from the corresponding author.
